# Safety culture among operating room healthcare workers: still a long way to go. An analytical cross-sectional study from Turkey*

**DOI:** 10.15649/cuidarte.2872

**Published:** 2023-09-09

**Authors:** Emel Filiz

**Affiliations:** 1 Assistant Professor of Selcuk University, Faculty of Health Sciences, Department of Health Management, Konya Turkey. E-mail: efiliz@selcuk.edu.tr Selçuk University Selcuk University Faculty of Health Sciences Department of Health Management Konya Turkey efiliz@selcuk.edu.tr

**Keywords:** Patient Safety, Culture, Hospital, Operating Room, Healthcare Workers, Benchmarking, Seguridad del Paciente, Cultura, Hospital, Sala de Operaciones, Trabajadores de la Salud, Evaluación Comparativa, Seguranga do Paciente, Cultura, Hospital, Sala de Operagáo, Profissionais de Saúde, Avaliagáo Comparativa

## Abstract

**Introduction:**

Safety culture attitudes of health workers are still not at the desired level. Although the creation of patient safety culture is important for all health care environments, it is more vital for critical units.

**Objective:**

To determine the patient safety culture levels of those working in the operating room environment and compare them with the 2008 results of the same hospitals.

**Materials and Methods:**

An analytical cross-sectional study was conducted in 2017-2018. The Turkish version of the Hospital Survey on Patient Safety Culture was administered to nurses, anesthesia technicians, assistant physicians, and specialist physicians working in the Operating Rooms (n=258) of two university hospitals in Konya, a large city in Anatolian region of Turkey.

**Results:**

Average percent positive response to the 42 items was low (41%, n=258). While there was no change in one dimension of the questionnaire compared to 2008; there was a positive change in 8 dimensions and a negative change in 3 dimensions. All 12 dimensions were lower than the Agency for Healthcare Research and Quality score.

**Discussion:**

Despite many studies, policy developments and interventions on patient safety, the improvement of a patient safety culture is very slowly in Turkey as in other countries.

**Conclusion:**

Non reporting of errors and a punitive approach in case of errors are still considered the most important problems.

## Resumen

**Introducción:**

las actitudes del personal de salud frente a la cultura de la seguridad siguen sin alcanzar su nivel deseado. Aunque la creación de una cultura de seguridad del paciente es importante en todos los entornos de cuidado, es vital en las unidades de cuidado crítico.

**Objetivo:**

determinar los niveles de cultura de seguridad del paciente de quienes trabajan en quirófanos y compararlos con los 2008 resultados de los mismos hospitales.

**Materiales y métodos:**

se realizó un estudio transversal analítico entre 2017 y 2018. La versión en turco de la Encuesta Hospitalaria sobre Cultura de Seguridad del Paciente se administró a profesionales de enfermería, anestesistas, médicos auxiliares y médicos especialistas que trabajaban en los quirófanos (n=258) de dos hospitales universitarios de Konya, una ciudad de la región de Anatolia en Turquía.

**Resultados:**

El porcentaje medio de respuestas positivas a los 42 ítems fue bajo (41%, n=258). Si bien no hubo cambios en una dimensión del cuestionario en comparación con los 2008 resultados, hubo un cambio positivo en 8 dimensiones y un cambio negativo en 3 dimensiones. Las 12 dimensiones presentaron una puntuación inferior a la de la Agencia para la Investigación y la Calidad del Cuidado de la Salud.

**Discusión:**

A pesar de los numerosos estudios, desarrollos en política e intervenciones en materia de seguridad del paciente, la mejora de la cultura de seguridad del paciente es muy lenta en Turquía, al igual que en otros países.

**Conclusión:**

No notificar errores y un enfoque punitivo en caso de error siguen considerándose los problemas más importantes.

## Resumo

**Introdujo:**

as atitudes de cultura de seguranza dos profissionais de saúde ainda nao estáo no nivel desejado. Embora a criagáo da cultura de seguranza do paciente seja importante para todos os ambientes de assisténcia médica, ela é mais vital para as unidades críticas.

**Objetivo:**

determinar os níveis de cultura de seguranza do paciente daqueles que trabalham no ambiente da sala de cirurgia e compará- los com os resultados de 2008 dos mesmos hospitais.

**Materiais e Métodos:**

um estudo analítico de corte transversal foi realizado em 2017-2018. A versáo turca da Pesquisa Hospitalar sobre Cultura de Seguranga do Paciente foi aplicada a enfermeiros, técnicos de anestesia, médicos assistentes e médicos especialistas que trabalham nas salas de cirurgia (n=258) de dois hospitais universitários em Konya, uma grande cidade na regiáo da Anatólia, Turquia.

**Resultados:**

a porcentagem média de respostas positivas aos 42 itens foi baixa (41%, n=258). Embora náo tenha havido alteragáo em uma dimensáo do questionário em comparagáo com 2008, houve uma alteragáo positiva em 8 dimensóes e uma alteragáo negativa em 3 dimensóes. Todas as 12 dimensóes foram inferiores a pontuagáo da Agency for Healthcare Research and Quality.

**Discussao:**

Apesar de muitos estudos, desenvolvimentos de políticas e intervengóes sobre a seguranga do paciente, o aprimoramento de uma cultura de seguranga do paciente é muito lento na Turquia, assim como em outros países.

**Conclusao:**

a náo notificagáo de erros e uma abordagem punitiva em caso de erros ainda sáo considerados os problemas mais importantes.

## Introduction

In the "To err is human" report of the US Institute of Medicine (IOM) in 1999^1^, the problem of unsafe health care services has attracted the attention of all societies after the declaration of high death rates due to health care services. Research results show that all rich and poor countries are affected by unsafe health care services today. According to a study, 60% of deaths related to health care services in middle- and low-income countries are caused by unsafe care, while the rest are related to access to health care. It is stated that the poor quality of health care is a greater obstacle to reduce the mortality rate than even insufficient access to health care^2^. Patient safety (PS) is not just a problem of poor countries. It is noted that one in 10 people in high-income countries suffers from poor-quality care when receiving medical care^3^.

Reporting of medical errors in Turkey started in 2016, but these reports do not contain official data on the outcome of patients. However, some data that can be considered as the tip of the iceberg can be accessed as some dramatic events are reflected in the press or are the subject of lawsuits. arikq et al. examined the medical malpractices (130 cases) that were reflected in daily newspapers in Turkey between 2015-2020^4^. Only 5% of cases were unharmed, 35% fatal, 28% unhealed, 14% disabled, and 19% unknown. In another study, 124 malpractice case files between the years 2015 2020 were examined^5^. In this study, it was determined that 49% of the patients could not recover, 27% disabled, 20% died, and 3% unknown. In these two studies, it is seen that medical errors are mostly experienced in operating rooms. Operating rooms (ORs), which are one of the most complex working environments of health organizations, are high-risk areas in terms of PS. About 7 million surgical patients suffer from significant complications annually due to unsafe surgical care procedures, while 1 million dies during or immediately after surgery^6^.

Ensuring the safety of patient care is possible by improving the safety culture in institutions. The Agency for Healthcare Research and Quality (AHRQ) defines Safety Culture as: “The safety culture of an organization is the product of individual and group values, attitudes, perceptions, competencies, and patterns of behavior that determine the commitment to, and the style and proficiency of, an organization's health and safety management”^7^. Countries and organizations can find their optimal ways to achieve a culture of safety. However, it should be known that there are some indispensable elements of the safety culture. Leadership commitment, transparency, openness to communication, learning from errors and best practices, and a reasonable balance between no-blame policy and accountability are indispensable components of safety culture^8^.

The starting point in establishing safety culture is the studies conducted to evaluate the current culture in the health care organization^9^. In recent years, we have seen an increase in the interest of health care organizations in evaluating the patient safety culture (PSC) using different measurement tools in many countries. In most of these studies, it has been shown that the safety culture attitudes of health workers are still not at the desired level^10^^-^^13^. In particular, staff shortages and non-punitive response to errors continue to be the most problematic areas of safety culture^14^.

The purpose of evaluating safety culture in institutions is to identify areas that need to be developed related to PS, raise awareness about PS in staff, monitor the change in PS initiatives over time, and allow internal and external comparisons of results^15^. The interest of researchers in the topic of PS in Turkey began about 14 years ago, and despite the growing interest, it seems that the topic has been studied within a relatively narrow scope^16^. The purpose of this study is to determine the PSC level of nurses, physicians, and other medical staff working in the ORs of two university hospitals in Konya province and compare them with the data of the 2008 studies^17^^-^^18^ and AHRQ 2018 database^19^.

## Material and Methods

### Study design and sample characteristics

This analytical cross-sectional study was conducted in two university hospitals located in the metropolitan center of Konya Province, Turkey. The HSOPSC user's guide recommends recruiting all staff if the survey is to be conducted in a specific unit of the hospital or units with less than 500 staff^7^. In addition, since it was hoped that the awareness of the personnel about patient safety would increase during the survey all OR healthcare workers (n=462) were requested to fill out the questionnaire. Staff who had been working in the OR for at least 6 months were included in the study.

### Research instruments

Data were collected using the Turkish version of the Hospital Survey on Patient Safety Culture (HSOPSC)^18^. Since the AHRQ published the Hospital Patient Safety Culture Survey in 2004, it has been used in hundreds of hospitals in the United States and around the world. This survey was also used to evaluate PSC in many hospitals in Turkey. HSOPSC includes 42 items (12 dimensions). Eighteen of the 42 items belonging to 12 dimensions were worded negatively. Survey item was measured on a 5-point Likert scale (strongly disagree to strongly agree and never to always). In addition, participants were asked to rate the area in which they worked in terms of patient safety and to indicate how many events they had reported in the last 12 months. In the last part of the survey, 5 questions were asked to the participants to provide background information about themselves.

### Data Collection

The data of the study were collected between December 2017 and February 2018. The purpose of the study and the privacy principles of the data were explained in the questionnaire form. Questionnaires were distributed to those who agreed to participate in the study and were collected at the end of the working day.

### Data analysis

The data were analyzed using SPSS (Statistical Package for Social Sciences) for Windows 26.0 program. Descriptive statistics of the sociodemographic and working environment characteristics of the participants were evaluated. Before analyzing the survey items, the negatively worded items were reverse coded. The evaluation of the data was carried out according to the 2018 user guide. For each positively worded item, the percentage of positive responses was calculated (agree and strongly agree or most of the time and always). The percentage of positive responses on all items included in the dimensions was averaged to calculate the score for each of the 12 safety culture dimensions.

One-way analysis of variance was used to compare PSC total score and sub-dimension scores among staff groups. Bonferroni-corrected P values were used as a post hoc test in the analysis of variance. The percentages of positive responses obtained from the survey were compared with the data obtained from the ORs-intensive-care and emergency units of Konya hospitals in 2008 and compared with AHRQ's 2018 database. P<0.05 was recognized as statistically significant. The database was stored in Mendeley Data^20^.

Ethical approval was obtained from Selcuk University Faculty of Health Science Ethics Committee for Non-Interventional Clinical Investigations (Date: 28.04.2017, No: 2017/26). In addition, permission from the hospital management and consent from the participants were obtained.

## Results

Participation rate in the study was 56% (258/462). Nurses had the highest rate among all participants (34.88% (90/258), followed by assistant physicians (34.50% (89/258), specialist physicians (17.83% (46/258), and anesthesia technicians (12.79% (33/258). Sixty-one percent of the participants were male (158/258). The mean age of the participants was 33.23± 8.42 years (min:20, max:58), and 47.29% of them were in the age range of 25-34 years. The rate of those with a professional experience of less than 5 years was 34.50% (89/258) and the rate of those with a working year in the OR of less than 5 years was 53.10% (137/258). Most of the participants stated that they worked more than 40 hours in a week (Table 1).

**Table 1:** Demographic and professional characteristics of the participants (N=258). Konya, 2017-2018. % (n).

Characteristics	Total	Nurses	Anesthesia technicians	Assistant Physicians (Surgery and Anesthesia)	Specialist Physicians (Surgery and Anesthesia)
	(n=258)	(n=90)	(n=33)	(n=89)	(n=46)
Gender					
Male	61.24 (158)	21.52 (34)	12.66 (20)	40.51 (64)	25.32 (40)
Female	38.76 (100)	56.00 (56)	13.00 (13)	25.00 (25)	6.00 (6)
Age (years)					
<24	12.79 (33)	42.42 (14)	54.55 (18)	3.03 (1)	0.00 (0)
25-34	47.29 (122)	22.95 (28)	4.10 (5)	67.21 (82)	5.74 (7)
35-44	29.46 (76)	50.00 (38)	13.16 (10)	6.58 (5)	30.26 (23)
<45	10.47 (27)	37.04 (10)	0.00 (0)	3.70 (1)	59.26 (16)
Experience in profession					
< 5 years	34.50 (89)	12.36 (11)	17.98 (16)	64.04 (57)	5.62 (5)
5-9 years	23.26 (60)	36.67 (22)	10.00 (6)	46.67 (28)	6.67 (4)
10-14 years	16.28 (42)	52.38 (22)	9.52 (4)	9.52 (4)	28.57 (12)
>15 years	25.97 (67)	52.24 (35)	10.45 (7)	0.00 (0)	37.31 (25)
Characteristics	Total	Nurses	Anesthesia technicians	Assistant Physicians (Surgery and Anesthesia)	Specialist Physicians (Surgery and Anesthesia)
	(n=258)	(n=90)	(n=33)	(n=89)	(n=46)
Experience in current hospital work area/unit					
<1 year	5.04 (13)	23.08 (3)	0.00 (0)	61.54 (8)	15.38 (2)
1-4 years	48.06 (124)	23.39 (29)	13.71 (17)	55.65 (69)	7.26 (9)
5-9 years	22.48 (58)	46.55 (27)	15.52 (9)	18.97 (11)	18.97 (11)
>10 years	24.42 (63)	49.21 (31)	11.11 (7)	1.59 (1)	38.10 (2)
Hours of work per week					
<40	11.63 (30)	63.33 (19)	16.67 (5)	3.33 (1)	16.67 (5)
41-49	38.76 (100)	57.00 (57)	16.00 (16)	7.00 (7)	20.00 (20)
>50	49.61 (128)	10.94 (14)	9.38 (12)	63.28 (81)	16.41 (21)

The positive response of the PSC was found to be low, with an average of 41% (106/258). Dimensions with the highest average percentage of positive responses were Teamwork within units (64% (165/258) and “Overall perceptions of safety” (59% (152/258). Dimensions with the lowest average percentage of positive responses were “frequency of events reported (18% (46/258)” and “non-punitive response to error (19% (49/258)”. The average positive response was higher in specialist physicians in seven of the 12 sub-dimensions than in other staff groups (Table 2).

**Table 2:** Positive response score of each dimension by occupation (% (n). Konya, 2017-2018.

Dimension	All Professionals (n=258)	Nurses (n=90)	Anesthesia technicians (n=33)	Assistant Physicians (AP) (Surgery and Anesthesia) (n=89)	Specialist Physicians (SP) (Surgery and Anesthesia) (n=46)	P-value*
Overall perceptions of safety	59 (152)	58 (52)	49 (16)	58 (52)	67 (31)	NS
Frequency of events reported	18 (46)	18 (16)	26 (9)	16 (14)	18 (8)	NS
Teamwork across units	43 (111)	44 (40)t	33 (11)t	35 (31)t	62 (29)	<0.01
Handoffs and transitions	46 (119)	48 (43)	40 (13)	45 (40)	47 (22)	NS
Management Expectations	41 (106)	41 (37)t	31 (10)t	33 (29)t	64 (29)	<0.01
Organizational learning	46 (119)	50 (45)	37 (12)t	39 (35)t	59 (27)	<0.01
Teamwork within units	64 (165)	60 (54)t	50 (17)t	65 (58)	81 (37)	<0.01
Communication Openness	35 (90)	36 (32)	29 (10)	31 (28)	43 (20)	NS
Feedback and communication about error	46 (119)	43 (39)t	45 (15)	40 (36)t	63 (29)	<0.01
Non-punitive response to error	19 (49)	15 (14)	16 (5)	22 (20)	20 (9)	NS
Staffing	29 (75)	26 (23)	21 (7)t	33 (29)	32 (15)	<0.01
Management support for resident safety	39 (101)	43 (39)t	25 (8)t	27 (24)§	62 (29)	<0.01
Overall	41 (106)	41 (37)t	34 (11)t	38 (34)t	53 (24)	<0.01

*Bonferroni-corrected P values in the analysis of variance. NS: not significant. t Lower than SP, $ Lower than AP, § Lower than SP and nurses

The present results were compared with the 2008 results of our hospital units (OR-emergency- intensive care). No change was found only in the dimension of "non-punitive response to error" (change: 0%). Three dimensions changed negatively. "Staffing" decreased from 37% to 29 % (change: -8%). "Handoffs and transitions" decreased from 50% to 46% (change: -4%). "Overall perceptions of safety" decreased from 60% to 59%. (change: -1%). Positive changes were observed in the remaining 8 dimensions. The biggest positive change was in the “feedback and communication about error” (change: 19%). Afterwards, there were positive changes in “management support for resident safety” (change: 9%); “communication openness” (change: 4%).

**Figure 1:**
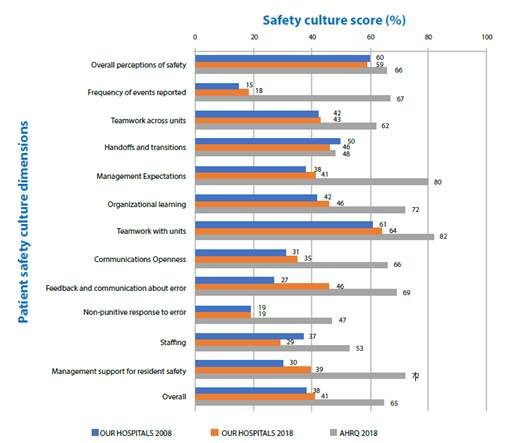
Comparing the mean scores of the operating room patient safety culture dimension with the 2008 mean scores of the same hospitals (Filiz, 2009) and the AHRQ benchmark scores (Famoloro et al.,2018). Konya, 2017-2018.

**Figure 2:**
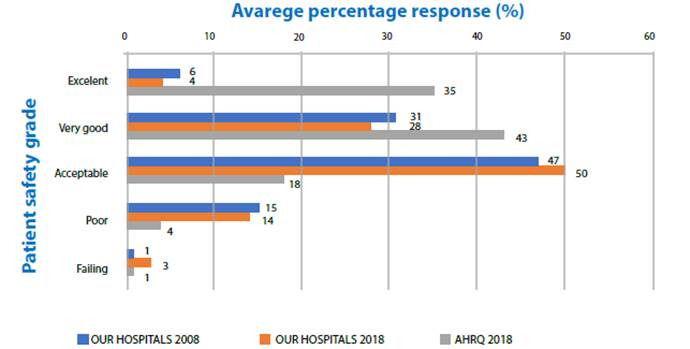
Comparing the percentage of participants giving their unit a patient safety grade with the 2008 data of the same hospitals (Filiz, 2009) and the AHRQ data (Famolaro et al.,2018). Konya, 2017-2018

**Figure 3:**
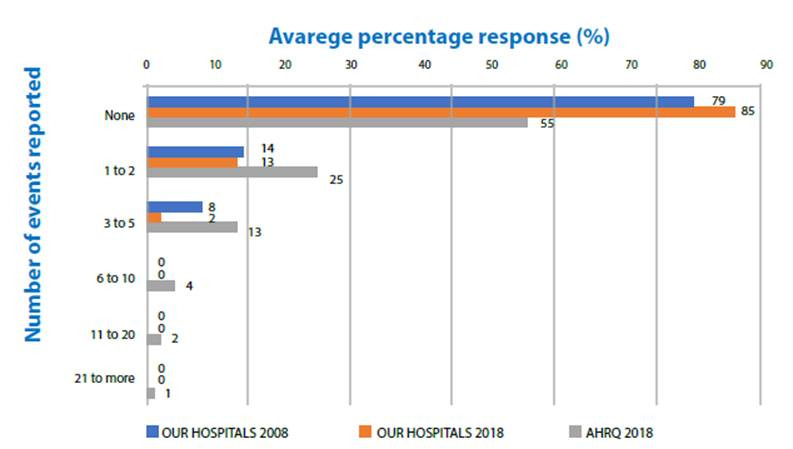
Comparing the percentage of participants reporting events in the past 12 months with the 2008 data of the same hospitals (Filiz, 2009) and the AHRQ data (Famolaro et al., 2018). Konya, 2017-2018

The change in other dimensions and overall score was 3%. The positive response scores of our hospitals for all 12 dimensions were lower than the AHRQ scores (Fig. 1). Positive response rates ("excellent" or "very good") to the PS grade decreased from 37% to 32% (change: -5%) (Fig. 2). The rate of respondents who reported one or more events over the past 12 months decreased from 22% to 15% (change: -7) (Fig. 3). Our scores were considerably lower than the AHRQ scores.

## Discussion

Although the creation of PSC is important for all health care environments, it is more vital for critical units such as ORs. In this study, PSC levels of nurses, specialist physicians, assistant physicians, and anesthesia technicians working in the OR were evaluated. Since they constitute the largest professional group working in hospitals, nurses had a higher proportion in this study than other team members as in similar studies^21^. In the study, the average positive response to PSC was found to be at a low level of 41%. In seven of the 12 sub-dimensions of the survey, the average positive response was found to be higher in specialist physicians than in other occupational groups (Table 2). Previous studies show that there is a difference in the perceptions of PSC among the types of staff working in hospitals^18^^,^^22^. It has been determined that physicians working in critical units evaluate the safety culture more positively than nurses^23^.

Conflicting results have been obtained in studies conducted in the perioperative area. In the Norwegian study^24^ while the anesthetists' perception of safety culture was more positive than that of surgeons and nurses, in other studies it was determined that surgeons' perception of safety culture was more positive than others^25^^-^^26^. A study conducted in the perioperative area showed that nurses and physicians' perceptions of PSC differed in some important dimensions^27^. Errors made in ORs where intensive technology and complex procedures are performed can lead to fatal consequences. The fact that OR and other critical unit staff have a safety culture that includes successful teamwork and communication skills depends on their perceptions, attitudes, knowledge, and skills in this regard^28^.

Results from this study were compared with our data from 10 years ago and AHRQ 2018 database. While there was no change in one dimension of the questionnaire compared to the previous study, there was a positive change in 8 dimensions and a negative change in 3 dimensions. The scores of all 12 dimensions were lower than the AHRQ score. The total positive response score (overall score) showed a positive change of 3% (2008: 38%; 2018: 41%; AHRQ: 65%) (Figure 1).

In the last 10 years in Turkey, similar problem areas have been identified in the studies conducted with AHRQ^18^. According to our findings, the areas with the highest scores were Teamwork within units (64%) and Overall perceptions of safety (59%). The most problematic areas that needed improvement were "frequency of events reported (18%)” and “non-punitive response to error (19%)”, and “staffing (29%)”. Our previous study^17^ showed that the PSC level was lower in the OR-Surgical Units-Emergency- Intensive Care units of hospitals. It has been suggested that the most common place for adverse events in hospitals is Ors^29^. Similarly, Wang and Tao^30^ compared the surgical units of hospitals with other units in his study and identified the dimensions of “teamwork within units” as strong, “staffing” and “non-punitive response to errors” as weak areas. In his study, 3 dimensions were weaker in surgical units than in other units. In Vlayen et al. study^31^, it was stated that the level of PSC was lower in the ORs and critical units.

The fact that there are some positive changes in our current study may reflect the studies conducted within the scope of quality in public hospitals. Quality studies in the field of health care services in Turkey have become systematic with the creation and implementation of “100 quality standards” in 2005. These standards prepared by the MoH have shown significant changes both numerically and within the scope over the years. The common point of the published standard sets has been to establish the quality culture at the basic level in health institutions and ensure their systematization. The implementation of these standards is mandatory for all hospitals in the country^32^. As a result of the policies developed by the Ministry of Health, the “Regulation on Ensuring Patient and Employee Safety^33^ and the “Regulation on the Development and Evaluation of Quality in Health” ^34^, it is likely that such legal regulations on PS mentioned in this study and other studies have contributed to the development of a safety culture.

In this study, the regression of the “staffing” dimension score by 8% is suggestive. Staffing is one of the most important factors affecting PS and is the most problematic area according to the results of the study35. The skilled nurse workforce has been shown to be associated with better patient outcomes, including lower hospital mortality^36^^-^^38^. In recent years, as a result of the health transformation policies of the Ministry of Health (MoH) with the new public management approach, contracted/subcontracted employment has been increasing instead of employing permanent nurses in health institutions^39^^-^^40^. Compared to the European Union and OECD countries, Turkey has the fewest healthcare workers per capita. Besides the insufficient number of nurses and physicians, their regional distribution is also an important problem. The majority prefer big cities instead of underdeveloped regions^40^. The country's policies indicate that underemployment will continue to be a problem in the future. However, it is suggested that the lack of care caused by staff shortages can be managed by implementing interventions that promote a positive work environment and a culture of PS^41^.

There was no change in the dimension of the “non-punitive response to error”in our findings. Fostering a culture of openness and learning from errors, rather than a culture of blame and punishment within the organization, is vital in raising the likelihood that staff will learn from errors^42^. Fear of punishment is one of the biggest obstacles to report incidents. Previous studies have shown that fear of punishment reduces reporting^43^^-^^45^. Health workers do not want the incident to arise with fears and concerns such as loss of reputation among colleagues, sometimes being punished or moving the incident to court. Hierarchy, competitive environments, and perfectionism, especially in traditional medical education, are serious obstacles to report medical errors^46^.

Although the “Frequency of events reported” field shows a positive change of 3%, it is still the most problematic area and is quite far from the AHRQ score (67%). Most often, employees of the medical institution do not report errors and do not share with others what they have learned from errors. As a result, the same errors occur repeatedly in many settings and patients continue to suffer from avoidable errors. As a solution to this problem, the health organization creates an effective reporting system at the regional or national level. Reporting can capture errors, injuries, non-harmful errors, equipment failures, process errors or other dangerous situations^47^. The work on creating a national “safety reporting system” (SRS) in Turkey began in 2016^48^. Laboratory errors account for 85% of the errors (101841 errors) reported to SRS in 2017^49^. It has been shown that error reporting is already a part of laboratory functioning processes as a reason for this. Therefore, only 15686 errors have been reported from other units and are far from reflecting the actual situation. It is noted that the most common type of error reported to SRS is the incorrect marking of the surgical site and patient falls.

The beginning of PS studies in Turkey dates to about 14 years ago^50^. Although many academic studies on patient safety have been conducted since then, clinicians' interest in the subject has not been at the same level. Despite numerous studies, policy reports and many interventions aimed at improving PS, progress is generally slower than originally envisaged^51^^-^^52^ and there are still problems that need to be overcome^53^.

### Limitations

Although the participants were assured that permission was obtained from the hospital administrators for this study, that personal information would not be shared with others, and that identity information would not be collected, most of the participants stated that they were hesitant to answer the questions, and therefore, the participation rate was low. Specialist physicians participated in the lowest rate. Those working in the operating rooms for at least 6 months were included in the sample. The high number of new hires due to the high turnover rate of non-physician personnel also caused the sample size to be small. Although AHRQ considers the participation of the staff at the rate of 30 50% sufficient, it states that the unit will be better represented with more participation.

There was also the possibility that responses of participants who felt under pressure may not have reflected their true feelings. This condition may affect the results of this study and may have shown the patient safety culture level differently than it was. Results of this study need to be supported by larger samples and multicenter studies. Another limitation of the study is the lack of institutional support for obtaining permissions for the survey and conducting the survey. This caused the data collection process to take longer than planned.

## Conclusions

The AHRQ HSOPSC questionnaire was used in this study, in which PSC was evaluated in the OR environment and compared with the results of 2008. While there was no change in one area of the survey, there was a positive change in 8 areas and a negative change in 3 areas. The scores of all 12 dimensions were lower than the AHRQ score. “non-reporting of errors” and "punitive response to error" were still identified as the most important problems.

Despite its limitations, this study has provided information about the improvement of patient safety culture in public hospitals and our position in international comparison, and also contributed to raising awareness about patient safety. The fact that the improve is slow in PSC shows the need for effective leadership. In order to improve PSC, it is especially important to eliminate staff shortages, create an environment where errors can be reported comfortably without worrying about being punished, and support staff for more effective and active use of the reporting system.

Because nurses constitute the largest group in healthcare and are in close contact with patients, their role in the development of a patient safety culture is critical. Nurse managers and healthcare leaders should continue to periodically use HSOPSC and similar surveys to identify areas for improvement and make comparisons across staff groups, units, hospitals and countries in future work to improve PSC. However, there is a need to know in more detail what the opportunities and barriers are for the development of a patient safety culture. Therefore, it will be useful to apply qualitative research methods in future studies. Qualitative studies can also provide useful information by determining the effectiveness of patient safety culture strategies on patient safety measures.
